# Polyphenols of Honeybee Origin with Applications in Dental Medicine

**DOI:** 10.3390/antibiotics9120856

**Published:** 2020-11-30

**Authors:** Carmen Curuțiu, Lia Mara Dițu, Alexandru Mihai Grumezescu, Alina Maria Holban

**Affiliations:** 1 Department of Microbiology and Immunology, Faculty of Biology, University of Bucharest, 030018 Bucharest, Romania; carmen.curutiu@bio.unibuc.ro (C.C.); lia-mara.ditu@bio.unibuc.ro (L.M.D.); alina.m.holban@bio.unibuc.ro (A.M.H.); 2 Department of Science and Engineering of Oxide Materials and Nanomaterials, Faculty of Applied Chemistry and Materials Science, University Politehnica of Bucharest, 1-7 Polizu Street, 011061 Bucharest, Romania; 3 Research Institute of the University of Bucharest—ICUB, University of Bucharest, 90-92 Panduri Road, 050657 Bucharest, Romania

**Keywords:** alternative antimicrobials, polyphenols, propolis, honeybee products, biofilm inhibition, dental diseases

## Abstract

Honeybee products are a great source of polyphenols with recognized applications in dental medicine. Although their biological mechanisms in oral diseases are not fully understood, numerous in vitro, in vivo and clinical studies have reported promising results in the prevention and treatment of oral diseases. Bioactivities, such as antibacterial, antiviral, antiparasite, anticancer, anti-inflammatory and anti-oxidant properties, recommend their future study in order to develop efficient alternatives in the management of widespread oral conditions, such as dental caries and periodontitis. The most investigated mechanisms of polyphenols in oral health rely on their ability to strengthen the dental enamel, decrease the development of dental plaque formation, inhibit the progression of dental caries and development of dental pathogens and show anti-inflammatory properties. These features recommend them as useful honeybee candidates in the management of emerging oral diseases.

## 1. Introduction

In the context of the current crisis in managing infectious diseases, alternative antimicrobial approaches are urgently needed [1,2,3].

Alternative antibiotics of natural origin, both plant and animal-derived, have increasingly attracted the attention of researchers in the last decade [4,5,6]. Honeybee products have proved their great biomedical potential since ancient times [7,8]. Their well-known curative and infection-preventive properties have been exploited in numerous applications. However, honeybee products may have significant differences in terms of chemical, physical, and biological composition, and such aspects could impact their biomedical properties [9,10]. For example, the composition of honey depends on its source (geographical area and plant species) and the genus and species of bees that produce the honey sample [11].

In order to overcome the limitations of natural products, identification of the most useful bioactive, and their dosage represents a critical step in the design of alternative antibiotics [12].

The most investigated honeybee products for their biomedical properties are: honey, beeswax, propolis, and royal jelly [13,14,15,16]. All these products may have diverse medical effects, being used today for preventing and healing severe diseases, such as cancers and infections. Moreover, these products are used on a wide scale in traditional treatments for increasing well-being and even life expectancy due to their proven antibacterial, antiparasitic, anticancer, anti-caries, and immunomodulatory potential [17]. Honey is mainly composed of sugars and water together with minor constituents such as minerals, vitamins, amino acids, organic acids, flavonoids, and other phenolic compounds and aromatic substances [18,19]. Beeswax consists of around 300 different compounds. These are mainly represented by long-chain alkanes, acids, esters, polyesters, and hydroxyl esters [20]. Propolis has a complex composition, being composed of about 58% phenolic compounds, 24% beeswax, 8% lipids and wax, 6% flavonoids, 0.5% terpenes, 0.5% bioelements and 3% other substances [21].

Royal jelly is a complex product, consisting of water (50–60%), proteins (18%), carbohydrates (15%), lipids (3–6%), trace minerals, water-soluble vitamins, free amino acids, and many other compounds, which are less known [13,22,23,24,25].

Although most of the major compounds found in honeybee products have proven biomedical potential, polyphenols have attracted the attention of researchers, mainly due to their wide distribution among all honeybee derived products (especially honey and propolis), but also because of their complex structure and properties (i.e., antibacterial, antiviral, anti-inflammatory, antineoplastic, and anti-ulcer effects) [26].

The biomedical potential of polyphenols also extends to dental applications. These natural origin compounds have been investigated for the improvement of dental materials and engineering of dental tissues [27]. Their antimicrobial potential may be used as a powerful approach in handling the biofilm of dental plaque, oral infections, and inflammatory conditions. In addition, antioxidant activities of polyphenols seem to offer significant protection against oral cancers [28], help in the management of the periodontal disease [29] and the prevention of dental caries and strengthen the teeth [30,31].

The purpose of this paper is to present the main types of polyphenols and discuss their applications in dental medicine, empathizing their antimicrobial effect and impact in the prevention and therapy of widespread dental pathologies.

## 2. Polyphenols in Honeybee Products

Polyphenols, the most numerous and widespread group of biologically active compounds, are secondary metabolites with a complex, heterogeneous chemical structure [32,33]. Based on the main monomer, the phenolic ring, polyphenols can be divided into two main classes: phenolic acids (hydroxybenzoic and hydroxycinnamic acids) and flavonoids (flavones, flavanones, flavonols, flavanols, and isoflavones) [31,34,35,36]. Lignans, stilbenes, and tannins are other important classes of polyphenols with different biological functions.

The phenolic content of honey is important for its biological effects. Different types of honey have different phenolic composition, depending on several factors, such as the bee species and the type of flowers from which they collect nectar, the geographical area, and the environmental conditions [37,38].

The polyphenol composition of honeybee products is very diverse and correlated to the geographical area of their production. Along with their high therapeutic and dietary value, polyphenols are primary identifying markers for the botanical origin of honey [39]. They are responsible for the colors, aromas, and tastes of all plant-based products, including fruits, cereals, and vegetables. Thus, the pollen source significantly impacts the color, aroma, and taste of honeybee products [26].

Flavonoids and phenolic acids are the most well-represented polyphenols in honeybee-derived products. The chemical structure of the bioactive flavonoid and phenolic acids in honey and propolis are largely similar across products originated in various geographic areas but vary in their relative quantities [40,41,42]. Generally, it seems that honey of darker color contains higher amounts of bioactive compounds as compared to lighter-colored honey [43].

For example, although there are qualitative and quantitative differences in flavonoid contents reported from different studies, probably based on the extraction and detection methods used, Manuka honey, monofloral honey derived from the manuka tree (*Leptospermum scoparium*) with proven antimicrobial and anti-oxidant properties is considered to be a better source of flavonoids than other types of honey. Pinobanksin, pinocembrin, and chrysin are the major flavonoids in Manuka honey, and in small quantities, luteolin, quercetin, 8-methoxykaempferol, isorhamnetin, kaempferol, and galangin. 4-Hydroxybenzoic acid and syringic acid are the most abundant phenolic acids detected in this type of honey [44].

In a study from 2019, Yi Tang et al. compared the content of polyphenols (16 phenolic acids and 14 flavonoids were investigated by HPLC) from 40 samples of honey from China and other countries [45]. Gallic acid, a well-known compound for the anti-oxidant activity of honey, was the most detected phenolic acid. In the respective study, almost all honey samples were found to be a good source of gallic acid except Loquat honey and Honeysuckle honey from China, in which gallic acid was not detected. In addition, chlorogenic acid and caffeic acid, which present very good antibacterial function, were investigated. Manuka honey from New Zealand presented the highest content of these two phenolic acids, followed by Forest honey from Spain and Wildflower honey from Italy. Vanillic acid was detected in Thyme honey from Spain, but not in Manuka honey, in which was detected *p*-hydroxybenzoic acid. Some other phenolic acids such as *p*-coumaric acid and syringaldehyde were detected only in two honey samples, whereas salicylic acid, sinapic acid, and ferulic acid were not detected at all. Among the flavonoids, apigenin and chrysin were most commonly found in honey from New Zealand and Spain. Significantly high content of quercetin was detected in Orange blossom honey. Chrysin, naringenin, and galangin were also found in Spanish honey samples, but not tangeretin and hesperidin. Based on these results, the authors conclude that the geographical location influences the honey composition in flavonoids. The floral origin determines the quantity of flavonoids [46].

This variation of the content of the polyphenols was also described by Olas Beata (2020) regarding the phenolic acid profiles of 12 kinds of honey collected from various regions in Greece: a significantly higher concentration of protocatechuic acid was detected in pine and fir honey than thyme and citrus honey, whereas *p*-hydroxybenzoic acid was the major phenolic compound detected in thyme honey [47].

Numerous studies performed to elucidate the most common bioactive flavonoid and phenolic acid compounds found in honeybee products have been demonstrated that they are: gallic acid, caffeic acid, ellagic acid, syringic acid, cinnamic acid, coumaric acid, benzoic acid (and its derivatives), quercetin, naringenin, kaempferol, luteolin, crysin and galancin [26,48].

## 3. Bioactivities of Polyphenols

Polyphenols have shown numerous mechanisms of biomedical interest, which could be exploited for their applications in dental medicine. Their anti-inflammatory, antimicrobial, anti-inflammatory, and tissue healing properties are currently being investigated for cardiac, digestive, and wound healing capacity. These mechanisms are very useful in the design of prophylactic and therapeutic approaches in oral pathologies, such as periodontitis (which has an important inflammatory component), oral cancers, and even dental caries. In the section below, we present a brief review of such useful properties.

### 3.1. Cardio-Protective and Anti-Ulcer Properties

Many of the phenolic constituents of honey-like quercetin, caffeic acid phenethyl ester (CAPE), acacetin, kaempferol, and galangin were found to have an encouraging effect in the prevention and treatment of cardiovascular diseases. These compounds showed antithrombotic, anti-ischemic, anti-oxidant, and vasorelaxant activity. The main activities of flavonoids included improving coronary vasodilatation and decreasing the blood coagulation capacity, and the oxidation of low-density lipoproteins (LDLs) [49].

Anti-ulcer activity is also attributed to flavonoids, such as kaempherol, quercetin, hesperitin, and naringin, which includes their capacity to stimulate the level of mucosal prostaglandins, and to decrease acid secretion and prevent ulceration [50]. There are also some in vitro studies that demonstrated the antimicrobial activity of different samples of honey against *Helicobacter pylori*, the bacterial species responsible for gastroduodenal ulcers [51].

### 3.2. Antitumor Activity

Besides other effects, honey also showed anti-tumoral activity by its capacity to induce apoptosis. It was demonstrated that honey inhibited colon cancer cell proliferation. The antimetastatic effect of propolis and some phenolic compounds was observed in tumor mice models before and after the injection of tumor cells. These effects were also reported for other types of cancer cell lines, like bladder cancer. Moreover, it seems that honey facilitated the antitumor activity of some chemotherapeutic agents such as 5-fluorouracil and cyclophosphamide. Researchers investigated if the anti-tumoral activity of honey could be attributed to its phenolic compounds. Many of them such as caffeic acid, caffeic acid phenyl ester, chrysin, galangin quercetin, acacetin, kaempferol, pinocembrin, pinobanksin, and apigenin were investigated and had an anti-proliferative effect in a dose-dependent manner in different types of cancers [52]. Phenolic compounds act on carcinogenesis through the induction of cell defense systems, including detoxifying and antioxidant enzyme systems, as well as the inhibition of the anti-inflammatory and anti-cellular growth signaling pathways that culminate in cell cycle arrest and/or cellular death. Polyphenols seem to elicit important alterations in the epigenome of cancer cells, which leads to a variety of anticancer mechanisms, including the modulation of cell cycle signaling, the removal of anticancer agents, the activity of antioxidant enzymes, apoptosis, and arrest of the cell cycle [53]. Polyphenols such as lycopene, epigallocatechin-3 gallate (EGCG), and sulphoraphane downregulate several signal transduction pathways responsible for anti-angiogenetic and antimetastatic activities, being efficient against prostate cancer [54]. EGCG can be methylated through catechol-O-methyltransferase and thus inhibit the DNA methyltransferase (DNMT), which prevents hypermethylation of newly formed DNA strands, leading to a reversal process of silenced genes. Furthermore, EGCG can suppress DNMT action by activating genes silenced by tumor cells by methylation. These mechanisms were demonstrated in colorectal cancer cells and are considered to also apply in other types of cancer cells [55]. EGCG and resveratrol decrease cell proliferation and induce DNA damage in various bladder tumor cells. The main incriminated mechanism is related to the modulation of the DNMT1 gene and apoptosis activation [56].

Resveratrol and quercetin are also active against human oral cancer cells. Studies demonstrated that 10 and 100 μM resveratrol induced significant dose-dependent inhibition in growth as well as in DNA synthesis of oral squamous carcinoma cells (SCC-25). Quercetin exhibits a biphasic effect, being simulative at 1 and 10 μM, while at 100 μM inhibits cell growth and DNA synthesis. Researchers combined 50 μM of resveratrol with 10, 25, and 50 μM of quercetin and demonstrated a gradual and significant increase in the inhibitory effect of quercetin on oral cancer cell growth and DNA synthesis [57]. These reports suggest that polyphenols and especially their combinations (such as resveratrol and quercetin) are effective inhibitors of oral squamous carcinoma cell growth and proliferation and warrant further investigation as cancer chemopreventive agents.

### 3.3. Antidiabetic Activity

Although honey has a large content of sugars, clinical studies revealed that the use of honey reduces the postprandial glycemic response, lowering the glucose serum concentration in patients with type 1 and type 2 diabetes. Because, in patients with diabetes, oxidative stress contributes to insulin resistance, a high proportion of LDLs are oxidized, and glycation determines endothelial damage, the use of honey is appropriate, taking into account its anti-oxidant capacity and its ability to inhibit lipid oxidation [58,59].

### 3.4. Neurological Diseases

In the last years, increased attention to the influence of diet and lifestyle on health has occurred and been demonstrated to have an important role in delaying the onset or stopping the progression of neurodegenerative diseases and also in improving cognitive function. Numerous studies have shown that the consumption of polyphenols decreased the risk of developing dementia and improved cognitive evolution, language, and verbal memory [60].

In neurodegenerative diseases such as Alzheimer’s disease or Parkinson’s disease, increased oxidative stress appears with the accumulation of reactive oxygen species (ROS), which are neurotoxic. Neuroinflammation, which is critical for the brain host defense, also contributes to the neuronal loss in these disorders and to damage associated with cerebral ischemia. In addition, the deposition of misfolded proteins, such as beta-amyloid, occurs. Polyphenols have the potential to protect neurons against injury induced by neurotoxins and have the ability to suppress neuroinflammation and counteract the pathological accumulation of proteins [61].

### 3.5. Wound Healing

Many studies have shown the great potential of polyphenols for wound healing due to their antioxidant, anti-inflammatory, and antimicrobial ability. The use of honey or propolis increases cellular proliferation and autolytic debridement and stimulates the immune system to reduce edema and pain.

### 3.6. Anti-Oxidant Activity

Anti-oxidants are substances that neutralize the effect of oxidants (such as free radicals or ROS). The absence of anti-oxidants determines the appearance of oxidative stress that can affect many physiological activities.

The polyphenols are considered the main compounds responsible for the antioxidant activity of honey. They show free radical scavenging activity, leading to the formation of more stable and less toxic molecules. They are hydrogen donors to free radicals from one of their hydroxyl groups [62,63]. In addition, they can inhibit oxidative processes by acting as lipoxygenase inhibitors and reducing agents for metmyoglobin [64]. The most investigated polyphenols in terms of anti-oxidant activity are curcumin and resveratrol [65]. The antioxidant properties of polyphenols are mainly mediated by their ability to down-regulate the nuclear factor NF-kB, modulating crucial cell signaling pathways involved in inflammation and cancer [66]. These natural compounds increase the antioxidant capacity of oral fluids, which suggests a preventive effect against periodontal disease [67], which is inextricably linked to oxidative-reductive (redox) imbalance. Due to the chronic inflammatory process, the total antioxidant capacity is reduced in gingival crevicular fluid and saliva of patients with periodontitis. Researchers state that such a condition may predispose to oxidative damage to proteins, lipids, and DNA and may cause progressive destruction of the periodontal attachment apparatus [68]. Honeybee derived polyphenols are potent antioxidants in both in vitro and in vivo biological systems. Such compounds extracted from honey, pollen, royal jelly, and propolis showed important antioxidant and free radical scavenging activities, as revealed through the 1,1-diphenyl-2-picryl hydrazyl (DPPH), ABTS (2,2′-Azinobis-(3-Ethylbenzthiazolin-6-Sulfonic Acid), Ferric Reducing Antioxidant Power (FRAP) and Oxygen radical absorbance capacity (ORAC) assays [69,70].

### 3.7. Antimicrobial Effect

Chemical structure can influence the antimicrobial activity of polyphenols. It seems that acids (i.e., hydroxycinnamic, phenolic, and hydroxybenzoic) have higher antibacterial activity as compared with their derivatives and other structures [71].

The following polyphenolic classes: stilbenes, cinnamic acids, benzoic acids, flavonoids, coumarins, and naphthoquinones, were intensively studied for their antimicrobial activity [72]. The molecular weight of polyphenols is very important for their antibacterial activity. Studies have reported that polyphenol concentrations equivalent to 1 g L^−1^ and 4 mmol L^−1^ cause a significant reduction in bacteria load in various opportunistic microorganisms [72].

In a study from 2004, the authors investigated the antimicrobial effect of 10 polyphenols (epigallocatechin (EGC), epigallocatechin-3 gallate (EGCG), punicalagin, tannic acid, castalagin, prodelphinidin, geraniin, procyanidins, a tea flavin mixture of black tea and green tea polyphenols treated with loquat polyphenol oxidase) against many strains of food-borne pathogens like *Staphylococcus aureus*, *Vibrio* sp., *Escherichia coli*, and *Salmonella*. The results revealed that many of the tested polyphenols had an antimicrobial effect, but the sensitivity of bacteria depends on bacterial species and also on polyphenol structure. EGC, EGCG, castalagin, and prodelphinidin oligomers showed relatively high antibacterial activities, suggesting the importance of 3,4,5-trihydroxyphenyl groups [73].

Gallic acid is a natural phenolic compound found in numerous fruits, medicinal plants, and honeybee products. The most relevant medical effects of gallic acid are anti-oxidant, anti-inflammatory, antimicrobial, and antineoplastic properties [74]. In vitro and in vivo studies have demonstrated that this polyphenolic compound has great therapeutic activities, being applied in inflammatory, gastrointestinal, metabolic, neuropsychological, urogenital, dermal, respiratory, cardiovascular diseases, malignancies, and oral health [74].

The antimicrobial activity of gallic acid is wide and proven on bacteria, fungi, parasites, and viruses [74]. This compound inhibits key virulence mechanisms, such as motility, adherence, and biofilm formation in clinically relevant gram-positive (i.e., *S. aureus*, *Streptococcus mutans*, *Listeria monocytogenes*) and gram-negative (i.e., *Pseudomonas aeruginosa*, *E. coli*, *Chromobacterium violaceum*, and *Campylobacter jejuni*) bacteria [75,76,77].

Ferulic acid, another phenolic compound, was proven to have antibacterial activity. It has also been shown that ferulic acid could be useful even in the treatment of some bacterial species known to be resistant to many antibiotics and difficult to treat, such as *Acinetobacter baumannii*. In this regard, it was observed that ferulic acid could enhance the efficiency of quinolone-based antibiotics against *A. baumannii* by increasing ROS generation [78].

Another study in which both gallic acid and ferulic acid were used demonstrated their antimicrobial properties against different species (*E. coli*, *P. aeruginosa*, *S. aureus*, and *L. monocytogenes*). The use of polyphenols is associated with irreversible changes in membrane properties that determine the formation of pores in the membrane and the leakage of cellular contents [71].

Quercetin, a 3,5,7,3′,4′-pentahydroxy flavone, is one of the most studied flavonoids, showing anti-oxidant, antithrombotic, anti-tumoral, antiviral, and antimicrobial properties.

Its antimicrobial activity was proven against *Bacillus subtilis, Micrococcus luteus*, and *Aspergillus flavus* but also against some *Staphylococcus* resistant strains. Moreover, it was demonstrated that quercetin had antibiofilm activity, 50% of the biofilm produced by *S. aureus*, and *S. saprophyticus* vancomycin-resistant strains being inhibited even at sub-inhibitory concentrations of quercetin [79].

Flavonoids, such as the compounds 2-phenyl acetophenone and trans-chalcone, showed enhanced anti-infective properties by inhibiting bacterial drug-efflux pumps and consequential synergism with antimicrobial agents. 2-phenyl acetophenone showed broad-spectrum efflux pump inhibition activity, whilst trans-chalcone displayed potent activity against Gram-negative (*E. coli*) and mycobacterial (*Mycobacterium smegmatis* ATCC700084, *Mycobacterium aurum* ATCC 10437, and *Mycobacterium bovis* BCG Pasteur ATCC 35734) efflux pumps. These polyphenols caused a higher inhibition than known potent efflux pump inhibitors, such as verapamil and chlorpromazine [80]. 2-phenyl acetophenone also showed the potential to work additively with known antibacterial agents that affect the cell-wall and DNA replication, while trans-chalcone has the potential to work synergistically with anti-tubercular agents [80].

Coumarin derivatives have also shown antimicrobial potential, being useful against Gram-positive and Gram-negative bacteria, as well as fungi. Coumarins showed minimum inhibitory concentrations (MICs) between 500 and 1000 µg/mL in fungal *Aspergillus*, *Penicillium*, and *Mucor* genuses and between 250 and 750 µg/mL in yeasts, such as *Candida albicans*. In bacteria, the MICs were 250–1000 µg/mL in both Gram-positive (ex. *Bacillus* sp) and Gram-negative (ex. *E. coli*) species [81]. In *Streptococcus* species, coumarins showed MICs of 0.9 to >12.4 μM [82]. Together with their wide antimicrobial potential, coumarins may interfere with bacteria Quorum Sensing (QS) signaling, and may control key mechanisms in dental pathologies, such as biofilm formation of pathogenic bacteria [83].

Coumarins of natural origin inhibited biofilm formation via QS regulation in *P. aeruginosa*, *E. coli*, and *S. aureus* strains [83].

## 4. Polyphenols in Dental Medicine

As potent antioxidants and antimicrobial agents, polyphenols of honeybee origin can help fight oral diseases due to their anti-inflammatory and anticarcinogenic actions. Products of natural origin are still the main source of health care treatment in some patients, and together with nutrition, play an important role in combating several diseases, including oral-derived ones.

Studies have demonstrated that polyphenols may be efficiently used as: (i) anti-inflammatory agents in the therapy of periodontitis [65], (ii) anti-proliferative compounds, being investigated for the prevention and therapy of oral cancers [84], (iii) antimicrobial agents for prevention and therapy of dental caries and also dental plaque biofilm inhibition [85], and (iv) repair materials of dental sockets [86,87].

Table 1 presents the most investigated types of polyphenols present in honeybee products for the prevention and therapy of dental diseases induced by microorganisms.

### 4.1. Dental Caries

The World Health Organization reports that nearly 100% of adults globally present with caries, as well as 60% to 90% of school-age children [102]. As a multifactorial disease, dental caries is an interplay between oral microbiota composition, the teeth particularities, and dietary factors. Polyphenols obtained from many natural products such as honeybee derivatives, tea, propolis, cranberry, *Galla chinensis*, grapes, coffee, and cacao proved efficient in the prevention of dental caries. Mouthwashes containing polyphenols are able to reduce the salivary level of bacteria species, which are responsible for caries development, such as *Streptococcus mutans* and *Lactobacilli* species [88]. They could also inhibit the growth, adherence, and acid production of the acidogenic oral *streptococci* [89]. In vitro studies have also demonstrated that polyphenols show powerful antimicrobial activity against planktonic and biofilm-embedded *E. faecalis.* Polyphenolic acid extracts from honey have impressive antimicrobial properties against *Streptococcus mutans* and *Rothia dentocariosa*, showed synergistic activity with antibiotics (i.e., amoxicillin), and are currently considered efficient nutraceutical agents to recommend in the prevention and treatment of oral diseases as a valid alternative to synthetic drugs [90].

The antimicrobial mechanism of polyphenols was intensively investigated, and results revealed that chemical structure might have an impact on the antimicrobial efficiency. For example, catechins produce irreversible damage to the microbial cytoplasmic membrane, control biofilm development and suppress multiple cariogenic virulence factors, mainly those associated with carbohydrate metabolism and acid tolerance, thus promoting the control of dental caries. Studies revealed that the expression of numerous genes controlling biofilm formation and production of soluble virulence factors, such as GtfB/C/D in *S. mutans* and *E. faecalis* is suppressed [91]. In addition, catechins also inhibit the activity of salivary amylase, leading to reduced cariogenicity of starch-containing foods [92].

Polyphenols in propolis have shown an inhibitory effect against the growth and the adherence of microbial species involved in caries development, such as *S. mutans*, *S. gordonii* [93], *Lactobacilli*, *Prevotella intermedia*, *Porphyromonas gingivalis*, *Actinomyces israelii*, and *Candida albicans* [94]. It seems that the complex bioactive mixtures present in honey (three types were investigated: Swiss midland honey, German lowland honey, and a Manuka honey) and propolis (utilized as 50% tincture in ethanol) inhibit the growth of oral pathogens and decrease the initial attachment of *S. gordonii*, while inhibiting demineralization of enamel by biofilm formation. Moreover, pellicle modification with the Swiss midland honey was protective against cariogenic surface hardness loss in vitro [93]. Such studies demonstrate that the source of honeybee products may be very important in dental caries applications. Propolis applied as ethanolic extract proved to be more efficient than chlorhexidine gluconate in inhibiting the planktonic growth of bacteria involved in dental caries and was also efficient against oral microorganisms in their biofilm state [94].

The topical application of propolis twice daily reduces the incidence and severity of dental caries in rats [103]. Compounds identified in propolis that belong to flavonoid aglycones, cinnamic acid derivatives, and terpenoids groups could inhibit either glucosyltransferases and/or growth of mutants streptococci at low concentrations. Initially, flavonoid and cinnamic acid derivatives were proposed as the most efficient propolis derived polyphenols against caries-producing bacteria [104]. However, recent studies have demonstrated that apigenin (4′,5,7-trihydroxylflavone) and trans-trans farnesol (tt-farnesol, 3,7,11-trimethyl-2,6,10-dodecatrien-1-ol) may exhibit significant biological activities against dental caries. Studies aiming to reveal their antimicrobial mechanisms showed apigenin effectively inhibits some virulence-related genes, such as GtfB and C in *S. mutans*, while tt-farnesol reduces cell viability by disrupting membrane integrity and destabilizing the oral biofilm [85,95]. Apigenin is a potent inhibitor of glucosyltransferases, but it has virtually no antibacterial activity against mutans streptococci. However, this polyphenol isolated from propolis regulated key virulence genes in *S. mutans* and thus controls its impact on caries development. tt-Farnesol showed bactericidal activity against planktonic cells and biofilms of mutans streptococci [105]. Anti-caries efficiency of tt-farnesol was also shown in vivo in a rat model. This polyphenol can reduce the severity of smooth surface caries in rats due to the lipophilic moiety interaction with bacterial membranes, being efficient also in the inhibition of *S. mutans* biofilms [96]. The data show clearly that apigenin (0.035%, *w*/*v*) and, to a lesser extent, tt-farnesol (0.028%, *w*/*v*) exhibit a cariostatic effect on smooth-surface caries even at low concentrations. Apigenin and tt-farnesol displayed distinct biological properties. The anti-caries mechanism of apigenin may be related to its exceptional inhibitory effects on glucosyltransferase activities both in solution and adsorbed onto saliva-coated hydroxyapatite beads. In addition, Gtf B and C are inhibited more than Gtf D by higher dilutions of apigenin. It seems that the deletion of genes controlling the production of glucosyltransferases, especially Gtf B and C, resulted in a dramatic decline in the virulence of *S. mutans* [106]. Apigenin effectively inhibited the activity of Gtf B and C enzymes and also showed cariostatic properties in the rat model [105]. The authors of the study suggest that apigenin affects the pathogenic potential of dental plaque related to caries by reducing the synthesis of extracellular glucans because it is devoid of antibacterial effects on mutants streptococci. This compound was reported as the most effective glucosyltransferase inhibitor tested so far, including currently commercially available mouth rinses [105].

Apigenin and tt-farnesol obtained from propolis are currently investigated for the development of alternative anti-caries products. Recent studies showed that topical applications (twice a day) of mixtures containing 1 mM apigenin, 5 mM tt-farnesol, and 13 mM fluoride significantly reduce the formation of *S. mutans* biofilms in rats [107]. After the application of this mixture in vitro, the amount of insoluble and iodophilic polysaccharides was drastically reduced in the biofilm, and the acidogenicity of the treated-biofilms was also reduced by 0.9–1.1 units. Moreover, the percentage of *S. mutans* UA159 (calculated from total cultivable oral microbiota and *S. mutans* population) recovered from the jaws of the rats after the treatment was significantly lower, as compared to the untreated control group [107]. The incidence of smooth-surface caries was reduced to up to 65% in treatment groups as compared with the control. The combination of these novel agents with fluoride may represent a potentially useful and alternative approach to the current chemotherapeutic strategies to prevent dental caries by reducing the expression of virulence of *S. mutans* without necessarily suppressing the resident oral microbiota.

A novel varnish containing apigenin and tt-farnesol from propolis and chitosan adheres to the tooth surface, quickly forms a film on the tooth surface, and continuously releases bioactive polyphenols for at least one week. This varnish was tested in vitro in bovine teeth and presented a tooth surface adherence and was able to form protective films very fast. The antimicrobial activity of the new propolis-based varnish against oral pathogens was shown to be higher than chlorhexidine varnish [97]. Authors of the study state that the obtained varnish could be translated into 5%, 10%, and 15% propolis content products, suitable for clinical application in the dental caries prevention field, deserving clinical studies to confirm its in vivo activity.

Other polyphenols, such as proanthocyanins, flavonols, and myricetin, can disrupt biofilm formation, inhibit attachment of oral pathogenic bacteria, and diminish the acidogenicity caused by *S. mutans* [98]. Such products are very efficient in reducing the development of carious lesions on the smooth surface and are promising novel alternatives or adjunctive anti-caries chemotherapy [85].

### 4.2. Periodontitis

Approximately 65 million individuals (almost half of the USA population aged 30 and older) have some type of periodontal disease [108]. Microbial subgingival colonizers responsible for periodontal destruction are: *Porphyromonas gingivalis*, *Prevotella intermedia*, *Fusobacterium nucleatum*, *Capnocytophaga* sp., *Lactobacillus* sp., *Treponema denticola* and *Tannerella forsythia* [99,109]. Results have demonstrated that polyphenols may have numerous activities for the prevention and treatment of periodontitis, both in vitro and in vivo. Their main activities are: (i) antimicrobial effects against subgingival colonizers, (ii) anti-inflammatory; (iii) modification of periodontitis severity parameters, such as probing depth (PD), gingival index (GI), and clinical attachment level (CAL) [65].

Main polyphenolic compounds obtained from honeybee products and other sources, such as hydroxybenzoic acids, hydroxycinnamic acids, hydroxyphenyl acetic acids, flavanols, flavanones, anthocyanins, flavones, isoflavonoids, and phenolics, have all been proven to inhibit periodontal pathogens under certain conditions. However, curcumin (followed by pyrogallol, pyrocatechol, and quercetin) was the most potent inhibitor of the growth of bacterial species responsible for periodontal destruction [99].

Moreover, these polyphenols proved to selectively target pathogenic biofilm microorganisms, especially *P. gingivalis*, while showing an insignificant impact on normal microbiota members of the dental biofilm such as *Streptococcus mitis* [99].

Since inflammation is a main trigger for periodontitis, studies have also focused on the impact of polyphenols in the modulation of inflammatory processes. It has been revealed that polyphenolic mixtures have significant anti-oxidant effects and may inhibit the release of several cytokines in vitro, in cultured human gingival epithelial cells [101].

In vivo polyphenol treatment applied in animal models has been shown to decrease inflammatory markers and macroscopic damage associated with periodontal disease. Curcumin was reported to decrease the circulating levels of IL-1β, TNF-α, and IL-17 inflammatory cytokines, which are recognized triggers of inflammation and disease progression in periodontitis [110]. Polyphenols can also reduce the extent of immune cell infiltration caused by bacteria into the periodontal tissues, which could help mitigate further inflammatory damage [65,111]. These bioactive compounds also modulate the expression of osteoclast-related genes, and reduce bone loss in mice [112]. Gelam honey (*Melaleuca cajuputi*) extracts have been shown to have a role in alveolar bone loss in experimental periodontitis, as showed in Sprague-Dawley rats. Hamzah and coworkers showed that systemically supplemented Gelam honey has the potential of reducing osteoclast activity in this experimental periodontitis rats, even though the effect on the alveolar bone level is not clear yet, and it warrants further research [113].

To our best knowledge, the polyphenols with the highest impact in decreasing the production of cytokines and in alleviating periodontitis dental changes, such as bone loss, are: myricetin, resveratrol, and curcumin [65,112].

Propolis polyphenols also proved to efficiently modulate the hallmarks of periodontitis in clinical trials. A hydroalcoholic solution of propolis extract applied twice a week for at least two weeks determined a significant decrease in the total viable counts of anaerobic bacteria (*p* = 0.007), an increase in the proportion of sites with low levels (≤10^5^ CFU/mL) of *Porphyromonas gingivalis* (*p* = 0.044), and an increase in the number of sites that are negative for bleeding on probing [114].

Moreover, propolis-based mouthwashes are very efficient in reducing plaque and gingival inflammation in clinical trials, as a recent study reveals [115]. Nine clinical trials comprising 333 human subjects were analyzed to demonstrate the efficacy of propolis mouthwashes compared with chlorhexidine, and the results clearly showed a higher efficiency of propolis on plaque and gingivitis inhibition or removal [115].

### 4.3. Dental Plaque

The biofilm of dental plaque is a multi-species and highly organized structure, which is mechanically and partially removed every day by personal brushing and formed again on the teeth surface. Briefly, the formation of the dental plaque biofilm follows the general biofilm formation steps: the formation of a conditioning film (acquired pellicle) on the surface of teeth and oral cavity, attachment of bacteria (early colonizers and then late colonizers), the maturation of the biofilm structure with the development of specific architectures and dissemination of cells embedded in biofilms [116].

Minutes after the tooth surface was cleaned, it rapidly gets covered with a glycoprotein layer (acquired pellicle), which is composed of salivary constituents, like albumin, lysozyme, amylase, immunoglobulin A, proline-rich proteins, and mucins. After the formation of the organic pellicle is completed, several microorganisms considered early (primary) colonizers attach to it. The most known primary colonizers of the dental plaque are Gram-positive bacteria belonging to the genus *Streptococcus* (*Streptococcus oralis*, *S. mutans*, *S. sanguis*, *S. mitis*, and *Actinomyces (Actinomyces viscosus)*. After the initial colonization, the plaque grows in size by the multiplication of already attached bacteria and by the attachment of new microbial species (secondary colonizers), which contain mainly Gram-negative species, such as *Fusobacterium nucleatum*, *Prevotella intermedia*, and *Capnocytophaga* sp. Further maturation of the dental plaque leads to the attachment of other species (late or tertiary colonizers), which attach to the already adhered Gram-negative species. They include *Porphyromonas gingivalis*, *Campylobacter rectus*, *Aggregatibacter actynomicetemcomitans*, and oral spirochete (ex. *Treponema denticola*) [116,117]. Along with these microbial species, studies have reported many other genera and species, which are considered very important in the development of dental plaque biofilm.

Polyphenols were shown to inhibit biofilm formation on tooth models in a dose and chemical composition manner. Polyphenolic mixtures used in low concentrations (0.16–0.31 mg/mL) inhibited biofilm formation without affecting the planktonic growth of dental biofilm species: *S. mutans* and *C. albicans*. Polyphenols inhibited the total cell growth by 54% and exopolysaccharide secretion by 81% in co-species biofilms of *S. mutans* and *C. albicans* [118].

Another study reveals that polyphenols applied together with an amine fluoride, Fluorinol(^®^), have enhanced antiplaque activity in vitro, being evaluated on a multi-species biofilm grown on saliva-coated hydroxyapatite discs. Biofilm formation was reduced by up to 4.76 log10, and this mixture inhibited insoluble glucan synthesis by glucosyltransferases by 97.4%. Moreover, this polyphenol–fluoride mixture showed very high anti-oxidant properties, even greater than vitamin C [119].

**Figure 1:**
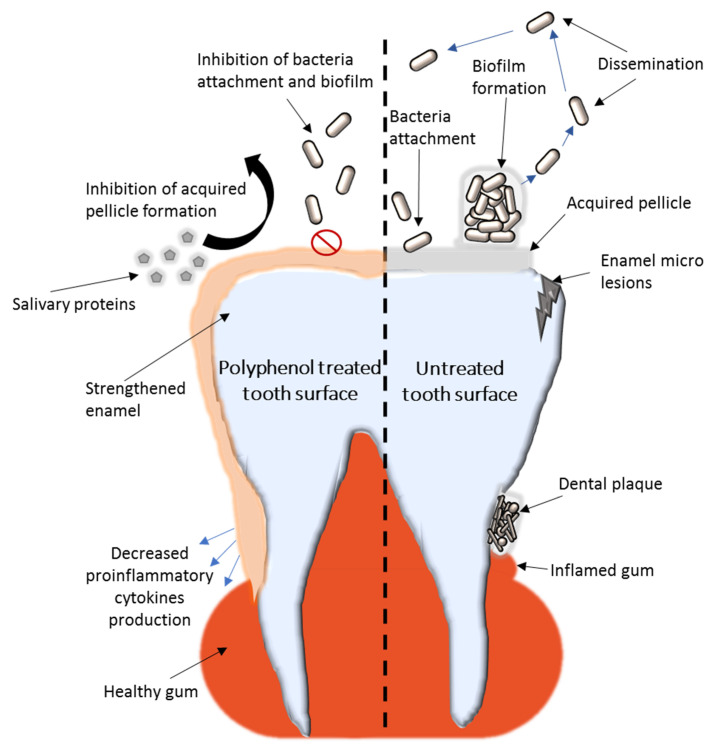
Representation of the known mechanisms of honeybee-derived polyphenols to inhibit dental plaque biofilm formation and dental caries.

A schematic representation of the known polyphenol mechanisms to prevent dental caries and plaque formation are presented in Figure 1.

### 4.4. Enamel Strengthening

Despite its low pH, honey does not cause enamel erosion. Oppositely, honey and propolis have been shown to offer enamel protection by inhibiting the attachment of degradative bacteria and even strengthen the enamel surface. The main enamel protection mechanism of honeybee based products seems to be related to enamel demineralization inhibition by the biofilm [77]. Polyphenols have been investigated recently for dentin bio-modification, offering promising perspectives in dental strengthening. Hydrolysable and condensed tannins, cardol and cardanol, have been used to achieve cross-linking in the dentin matrix. After only 1 min of treatment, the best bio-modifiers were cardol and cardanol [120]. Recent studies reported that the formation of polyphenol-induced cross-links in the collagen matrix provides cohesion and makes it more resistant to degradation. Tannic acid was shown to form stable cross-links with exposed collagen fibrils, allowing them to increase the resistance against their degradation process. These compounds increase the stiffness of demineralized dentin and reduce the enzymatic degradation of collagen. The proposed mechanism is related to hydrogen bonds between the biopolymer and tannic acid [121]. It was also shown that tannic acid incorporated into polycarboxylate cement enhances the resistance of dentinal collagen to collagenase and proteolytic enzymes.

Gallic acid is able to reduce dentin fluid flow, which is the main cause of dentin hypersensitivity. The formed catechol-iron complex is deposited on the surface, creating stable cross-linked complexes in the oral aqueous environment, creating tight bridge-like connections between adjacent peritubular dentin, which resulted in less outward flow. In addition, a complex made of fluoride-tannin acid-lanthanum-apatite was also reportedly used to reduce dentinal hypersensitivity by similar mechanisms [27,122].

### 4.5. Oral Cancers

Honeybee-originated polyphenols have also shown enhanced anticancer properties, being efficient against initiation, proliferation, and progression of oral cancers. Their main anticancer mechanisms refer to the induction of apoptosis, cell cycle arrest, the modulation of oxidative stress, the amelioration of inflammation, the induction of mitochondrial outer membrane permeabilization, and the inhibition of angiogenesis [123].

Tualang honey showed anti-proliferative and proapoptotic effects on oral squamous cell carcinoma in vitro [124]. The antitumor activity of crude honey extracts in oral cancers seems to be related to the induction of apoptosis through caspase 3 activation [52]. Due to well-developed purification techniques, in recent years, cancer research has focused on purified polyphenols instead of crude honey. The most investigated simple polyphenols in honeybee products that have evolved as promising pharmacological agents in the treatment of cancer are caffeic acid, caffeic acid phenyl esters, chrysin, galangin, quercetin, kaempferol, acacetin, pinocembrin, pinobanksin, and apigenin [52]. Although these phenolic compounds showed significant anticancer properties in colon cancer, gastric cancer, skin cancer, fibrosarcoma, and glioma cell cancer, research regarding the mechanisms of oral cancer inhibition is widely unavailable. Due to their easy application on the oral cavity, proven anticancer impact in other cancers, and diversity of the honeybee polyphenolic compounds, these offer an impressive perspective in developing an efficient, natural, non-toxic, and personalized alternative in the therapy of oral cancers [52].

**Figure 2:**
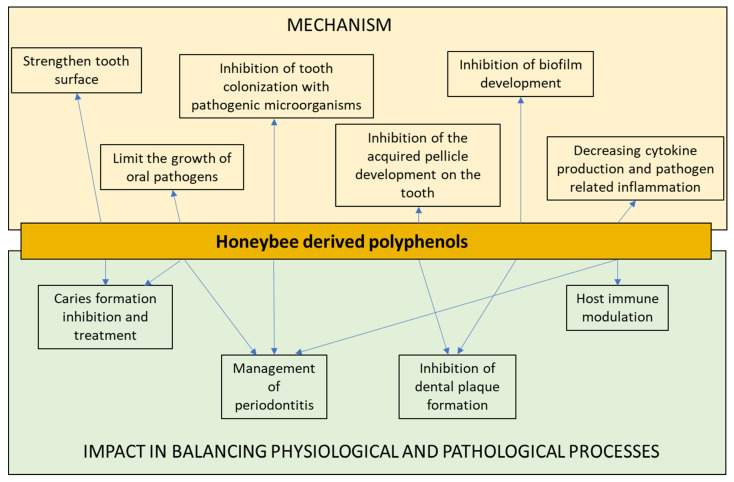
The main mechanisms of honeybee polyphenols useful in oral medicine and their consequence in balancing oral health and microbial-related disease. The upper part of the scheme represents the known mechanisms of polyphenols, which can be exploited in balancing oral health and disease (possible consequences are represented in the lower part of the scheme). Widespread diseases, such as dental caries could be prevented or treated by the application of polyphenol-based products, which strengthen the tooth surface and limit the growth of oral pathogens. These compounds may have an impact on the management of oral multifactorial conditions such as periodontitis by exploiting their ability to inhibit the attachment, growth, and biofilm formation of pathogenic bacteria and also by modulating inflammation at the affected site.

Figure 2 shows the main processes and mechanisms induced by polyphenols in balancing oral health and disease.

## 5. Conclusions

Natural products, such as honeybee-derived products, contain numerous bioactive compounds with impressive biomedical potential. Polyphenols are currently investigated in order to develop new alternatives in the treatment of severe health conditions, such as infections and cancer. The use of these versatile bioactive compounds in oral health is increasing. In recent years researchers have made important progress in elucidating mechanisms of polyphenols that could be exploited for dental medicine.

Their antioxidant, anti-inflammatory, and antimicrobial effects are considered the most important traits to recommend these classes of compounds in oral health. Polyphenols found in honeybee products can be easily obtained and safely utilized for daily care, as demonstrated by several in vitro, in vivo, and clinical studies. They could reduce the risk of emerging oral diseases such as dental caries and inflammatory conditions, which have a microbial component, such as periodontitis. Moreover, researchers are making constant progress in elucidating their anti-tumoral mechanisms; therefore, polyphenols offer great perspectives in treating oral cancers.

## Figures and Tables

**Table 1:** List of the main polyphenols investigated for their impact in the prevention and therapy of oral dental pathologies containing a microbial component.

Oral Pathology	Type of Polyphenols	Antimicrobial Mechanism	Microbial Pathogen	Reference
***Dental caries***	mixture of polyphenols	inhibition of bacterial growth	*Streptococcus mutans* and *Lactobacilli* species	[88]
		inhibition of bacterial growth, adherence, and acid production	acidogenic oral *streptococci*	[89]
	polyphenolic acid extracts form honey	growth inhibition	*Streptococcus mutans* and *Rothia dentocariosa*	[90]
	catechins	suppression of GtfB/C/D genes (responsible for biofilm formation and production of soluble virulence factors)	*S. mutans* and *Enterococcus faecalis*	[91]
	catechins	inhibit the activity of salivary amylase and bacterial attachment	inhibits adherence of dental colonizers	[92]
	Mixture of polyphenols from propolis	inhibition of bacterial growth and adherence	*S. mutans* and *S. gordonii*	[93]
			*Lactobacilli*, *Prevotella intermedia*, *Porphyromonas gingivalis*, *Actinomyces israelii*, and *Candida albicans*	[94]
	apigenin	inhibit some virulence-related genes, such as GtfB and C	*S. mutans*	[85,95]
	trans-trans farnesol	reduce cell viability		
	trans-trans farnesol	inhibition of biofilms	*S. mutans*	[96]
	apigenin and tt-farnesol from propolis	inhibit bacterial growth and biofilm	Biofilm oral pathogens (bulk)	[97]
	proanthocyanins, flavonols, and myricetin	disrupt biofilm formation, inhibit attachment, and diminishes the acidogenicity	*S. mutans*	[98]
***Periodontitis***	curcumin (followed by pyrogallol, pyrocatechol, and quercetin)	inhibition of growth	*P. gingivalis*	[99]
	epigallocatechin-3-gallate	growth inhibition Inhibition of cytokine production in a host	*S. mutans*	[100,101]
